# Comment on: Association between the beta‐blockers, calcium channel blockers, all‐cause mortality, and length of hospitalization in patients with heart failure with preserved ejection fraction: A meta‐analysis of randomized controlled trials

**DOI:** 10.1002/clc.24125

**Published:** 2023-09-19

**Authors:** Vadim Zakiev, Anna Gvozdeva, Anton Skotnikov

**Affiliations:** ^1^ Russian Clinical and Research Center of Gerontology Pirogov Russian National Research Medical University Moscow Russia; ^2^ Chazov National Medical Research Center of Cardiology Myasnikov Institute of Clinical Cardiology Moscow Russia; ^3^ Department of Medical and Social Assessment, Emergency, and Ambulatory Practice Sechenov First Moscow State Medical University Moscow Russia

To the Editor,

The article by Wu et al.^1^ published in June 2023 in the “Clinical Cardiology” discuss an actual issue of treatment of Heart Failure with preserved left ventricular ejection fraction. The authors performed a meta‐analysis and concluded that in comparison with a placebo, both beta‐blockers (BB) and calcium channel blockers (CCB) substantially reduced the risk of all‐cause mortality and hospitalization. However, BB are more effective because they provide a significant reduction in all‐cause mortality and hospitalization as compared with CCB. First, we would like to thank the authors for such an important article. However, there are some issues in the article.

The authors stated that they had performed a meta‐analysis of randomized controlled trials (RCT) while some of the studies included were not RCT. For example, the study performed by Gomez‐Soto et al.^2^ was case–control population study, and the article by Patel et al.^3^ is based on the US national real‐world registry OPTIMIZE‐HF. In our view, it is important to separate observational studies and RCT as the population may differ. Real‐world patients usually have more comorbidities, which can affect the result. The previously published meta‐analysis showed different results for observational studies and RCT.^4^ We are familiar with some other relevant RCT's^5^, ^6^ and it is unclear to us why they were not included in this meta‐analysis. We respectfully ask Wu et al. to further elaborate their retrieval strategies.

Finally, there are some mistakes in the tables. The references numbers 19 and 20 are confused in table 1 and figures 2 and 3. The sample size, drug, or intervention seems to be confused in table 1. In addition, reference number 20 is a rational and design publication, but not a publication of the results of the study.^7^, ^8^

We suppose that the issues discussed above might mislead the reader. We hope to discuss them with the authors.

## References

[1] Wu M , Ni D , Huang L , Qiu S. Association between the beta‐blockers, calcium channel blockers, all‐cause mortality and length of hospitalization in patients with heart failure with preserved ejection fraction: a meta‐analysis of randomized controlled trials. *Clin Cardiol*. Published online June 4, 2023. 10.1002/clc.24058 PMC10436801 37272188

[2] Gomez‐Soto FM , Romero SP , Bernal JA , et al. Mortality and morbidity of newly diagnosed heart failure with preserved systolic function treated with β‐blockers: a propensity‐adjusted case‐control populational study. Int J Cardiol. 2011;146(1):51‐55. 10.1016/j.ijcard.2009.06.009 19573938

[3] Patel K , Fonarow GC , Ekundayo OJ , et al. Beta‐blockers in older patients with heart failure and preserved ejection fraction: class, dosage, and outcomes. Int J Cardiol. 2014;173(3):393‐401. 10.1016/j.ijcard.2014.03.005 24703206 PMC4085276

[4] Fukuta H , Goto T , Wakami K , Ohte N . The effect of beta‐blockers on mortality in heart failure with preserved ejection fraction: a meta‐analysis of observational cohort and randomized controlled studies. Int J Cardiol. 2017;228:4‐10. 10.1016/j.ijcard.2016.11.239 27863360

[5] van Veldhuisen DJ , Cohen‐Solal A , Böhm M , et al. SENIORS Investigators Beta‐blockade with nebivolol in elderly heart failure patients with impaired and preserved left ventricular ejection fraction. JACC. 2009;53(23):2150‐2158. 10.1016/j.jacc.2009.02.046 19497441

[6] Silverman DN , Plante TB , Infeld M , et al. Association of β‐Blocker use with heart failure hospitalizations and cardiovascular disease mortality among patients with heart failure with a preserved ejection fraction: a secondary analysis of the TOPCAT trial. JAMA Netwk Open. 2019;2(12):e1916598. 10.1001/jamanetworkopen.2019.16598 PMC6902757 31800067

[7] Palau P , Seller J , Domínguez E , et al. Beta‐blockers withdrawal in patients with heart failure with preserved ejection fraction and chronotropic incompetence: effect on functional capacity rationale and study design of a prospective, randomized, controlled trial (The Preserve‐HR trial). Clin Cardiol. 2020;43:423‐429. 10.1002/clc.23345 32073676 PMC7244302

[8] Palau P , Seller J , Domínguez E , et al. Effect of β‐blocker withdrawal on functional capacity in heart failure and preserved ejection fraction. J Am Coll Cardiol. 2021;78(21):2042‐2056. 10.1016/j.jacc.2021.08.073 34794685

